# Dr. Wellington Prosser

**Published:** 1912-10

**Authors:** 


Dr. Wellington Prosser, a retired physician of Olean, died suddenly of apoplexy, Aug. 24, aged 78.

